# Research on Pedestrian Crossing Decision Models and Predictions Based on Machine Learning

**DOI:** 10.3390/s24010258

**Published:** 2024-01-01

**Authors:** Jun Cai, Mengjia Wang, Yishuang Wu

**Affiliations:** School of Architecture & Fine Art, Dalian University of Technology, Dalian 116024, China; caimans@dlut.edu.cn (J.C.); wuyishuang@mail.dlut.edu.cn (Y.W.)

**Keywords:** intelligent traffic safety, machine learning, pedestrian crossing, characteristics of crossing behavior, available crossing gaps

## Abstract

Systematically and comprehensively enhancing road traffic safety using artificial intelligence (AI) is of paramount importance, and it is gradually becoming a crucial framework in smart cities. Within this context of heightened attention, we propose to utilize machine learning (ML) to optimize and ameliorate pedestrian crossing predictions in intelligent transportation systems, where the crossing process is vital to pedestrian crossing behavior. Compared with traditional analytical models, the application of OpenCV image recognition and machine learning methods can analyze the mechanisms of pedestrian crossing behaviors with greater accuracy, thereby more precisely judging and simulating pedestrian violations in crossing. Authentic pedestrian crossing behavior data were extracted from signalized intersection scenarios in Chinese cities, and several machine learning models, including decision trees, multilayer perceptrons, Bayesian algorithms, and support vector machines, were trained and tested. In comparing the various models, the results indicate that the support vector machine (SVM) model exhibited optimal accuracy in predicting pedestrian crossing probabilities and speeds, and it can be applied in pedestrian crossing prediction and traffic simulation systems in intelligent transportation.

## 1. Introduction

The current paradigm in road design significantly prioritizes motor vehicles, rendering pedestrians in a vulnerable position and maintaining a high rate of pedestrian accidents. As per the *2021 China Traffic Accident Statistical Yearbook*, motorized, non-motorized, and pedestrian accidents were recorded at 2.67 million, 390,000, and 140,000 incidents, respectively. Hence, understanding pedestrian crossing behaviors and decision-making processes is crucial. Although several methods such as the Harders, Ashworth, and Raff methods, as well as the Logit process calculation and the maximum likelihood estimation method, have been widely researched for investigating vehicle crossing gaps, there is a notable deficit in studies focusing on pedestrian crossing decision making. Some scholars have suggested calculating critical gaps using the average single-lane pedestrian crossing time. However, the randomness in pedestrian crossings introduces errors to this method. Furthermore, pedestrian crossing resembles a dynamic game, where both vehicles and pedestrians aim to traverse with minimum delays and the utmost safety, but due to the unpredictability of choices, not all pedestrians choose to cross even with ample gaps. A dynamic game theory perspective has revealed that a pedestrian’s decision to cross is influenced by the interacting vehicle’s speed and distance or the headway time. Presently, research on pedestrian crossing decision making primarily revolves around two aspects, which are discussed below.

The first facet involves exploring pedestrians’ permissible crossing gaps. Early research, such as that by Wilson and Grayson [1], primarily analyzed the proportion of acceptable gaps for various demographic groups, revealing, for instance, that 11.1% of pedestrians accepted a crossing gap of less than 2 s. Chae et al. [2] discerned an acceptable pedestrian crossing gap of approximately 5.1 s using data from American roundabouts. Meanwhile, subsequent gap research, such as the studies by Himanen [3] and Cavallo [4], have primarily concentrated on formulating crossable gap models and dissecting influencing factors, such as vehicle speed, distance, and pedestrian age, all of which are pivotal in determining gap acceptance. Further studies have explored various pedestrian behaviors. Kadali and Rathi [5] illustrated the notable impacts of pedestrian crossing speed and conflicting vehicle speed and distance on gap acceptance. Shabban and Mohammed [6] developed models for two street-crossing methods, revealing that factors such as crossing distance and pedestrian crossing speed significantly influenced both. A distinct relationship between young pedestrians and accident proneness was identified by Niaz et al. [7], while Granié [8] and Ezzati Amini [9] highlighted that younger pedestrians often engage in riskier crossing behaviors and accept smaller gaps. Contrarily, Dommes et al. [10] emphasized elderly pedestrians’ higher collision risks due to attention and physical limitations. Zafri et al. [11] showcased that elderly individuals exhibit fewer rolling gap crossings, underlining varied findings across studies on different age demographics and their crossing behaviors.

The second domain involves researching pedestrian crossing speeds, a critical parameter in intersection design that ensures both safety and traffic efficiency. The initial studies on this subject predominantly analyzed the influences of age and gender on crossing speeds. Govinda L. and Abhigna D. [12] demonstrated that young individuals generally traverse faster than the elderly, with gender presenting no substantial impact on crossing speed. Moore [13] identified that smaller accepted gaps were correlated with faster crossing speeds (i.e., 1.2 m/s for gaps of > 7 s and 1.5 m/s for those of < 3 s). In subsequent research, Lam and Lee [14] found that the average speed during red-light crossings was 1.5 m/s, which exceeded the 1.27 m/s observed during green-light crossings. Similarly, Gates and Noyce [15] found a higher average speed during non-green-light crossings (1.57 m/s) compared with green-light crossing speeds (1.37 m/s). Recent findings by Koh and Wong [16] have highlighted significant differences between walking and cycling crossing speeds. Feng Shumin and Wu Yuexin [17] deduced an average pedestrian crossing speed of 1.47 m/s, with an 85th percentile of 1.74 m/s, using data from Harbin. By examining data from Nanjing’s Xinjiekou area, Lu Jian and Ye Huiqiong [18] established elderly and young people’s average crossing speeds as 1.17 m/s and 1.29 m/s, respectively. Figueroa-Medina et al. [19] pinpointed age and acceptable gap as the significant determinants of pedestrian crossing speed. Ku et al. [20] employed a discriminative algorithm based on deep image learning to conduct a quantitative analysis of the safety and economic issues arising from traffic vulnerability. Their study did not consider the exact number of dispatchers required for certain aspects of deep learning and the data collection process. Alver et al. [21] proposed a comprehensive AHP-FL (analytic hierarchy process–fuzzy logic) method to address the issue of how pedestrians assess the safety of available gaps. However, this method is susceptible to individual subjective biases. Li et al. [22] introduced a method combining foreground detection with deep learning to detect moving pedestrians, effectively utilizing the invariant background of video images.

However, the existing research faces the following issues: (1) traditional models, primarily those using non-trajectory data, inadequately illustrate the dynamic and mobile characteristics impacting pedestrian crossing decisions; (2) most studies on pedestrian crossing gaps have focused on predicting the psychological thresholds for crossing without adequately simulating actual behaviors. Using image recognition and machine learning can address these problems, enabling more accurate analysis of factors affecting unlawful pedestrian crossings, thereby optimizing decision models and precisely simulating crossing behaviors. Machine learning methods offer the ability to efficiently process and analyze large volumes of complex data, adapt and improve over time, automate decision-making processes, and provide powerful predictive and personalization capabilities across various domains. One’s suggestions make the logic more standardized. This study first synthesized the existing research, and the first chapter of this paper discusses the OpenCV-based data collection and extraction, which we applied to five Dalian City intersections. The second chapter presents our methodology, which used various predictive methods. The third part validates and compares each method, and it conducts a feature analysis. The fourth part outlines our conclusions and projections.

## 2. Transportation Surveys and Data Processing

### 2.1. Survey and Sampling

To ensure sample diversity, this study selected four representative intersections in Dalian, China. Intersection 1 was in an aging residential area with many elderly residents. Intersection 2 was surrounded by modern commercial complexes. Intersection 3 was near primary and secondary schools. Intersection 4 was near a newly developed residential area with nearby amenities such as a small plaza and kindergarten. These four intersections were chosen to guarantee the data comprehensiveness and accuracy.

#### 2.1.1. Location

**Figure 1:**
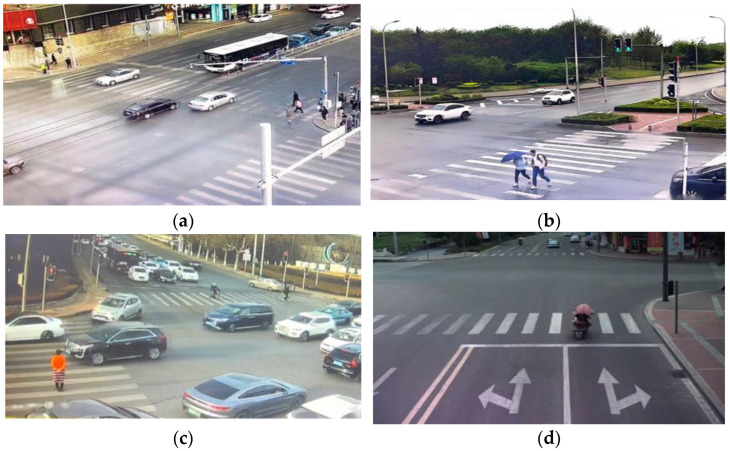
Camera angles at four data collection sites: (**a**) Shandong Road–Songjiang Road; (**b**) Hongyun Road–Zhelin Street; (**c**) Zhangqian Road–Hongjin Road; and (**d**) Huadong Road–Qianshan Road.

Data were gathered using wide-angle cameras, with a total of four wide-angle cameras and four roadside cameras strategically installed across four collection sites, as shown in Figure 1. This setup ensured that the pedestrian and vehicle data collected at these locations were sufficiently clear and abundant for analysis.

#### 2.1.2. Collection Time

Video data for this study were collected during busy morning and evening peak hours, characterized by high vehicle flow and low speeds, alongside increased pedestrian crossing demands. Additionally, data were gathered during less busy off-peak periods with lower vehicle flow and higher speeds, resulting in fewer pedestrian crossings. This method ensured data accuracy and completeness. Collection dates and durations are detailed in the accompanying table. Cameras were discreetly set up to avoid influencing pedestrian behavior, thus maintaining the accuracy and authenticity of the data. The video collection times and durations for each surveyed road section are listed in the Table 1 below.

**Figure 2:**
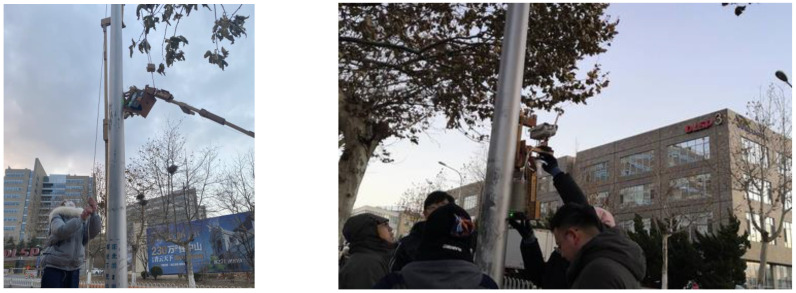
Installation process of cameras for data collection.

The lengthy and high-resolution videos collected consumed substantial memory. To protect pedestrian and driver privacy and manage memory use, the resolution was reduced, ensuring that vehicle and pedestrian data remained identifiable. Cameras were suspended at an elevated position on electric iron poles on both sides of the road using pulleys. They were secured in place via magnetic attachment, as shown in Figure 2. The collection team remotely controlled the camera’s shooting direction and tilt angle using a motorized pan-tilt head, ensuring that the camera’s field of view adequately covered the required areas.

### 2.2. Data Collection Methods and Numerical Statistics Based on OpenCV

**Figure 3:**
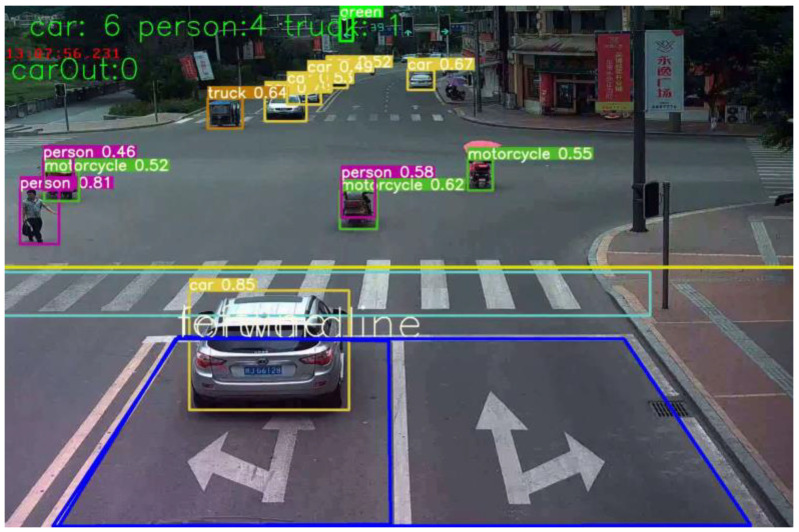
Image recognition interface.

Surface traffic data surveys fundamentally inform our understanding of pedestrian crossing and vehicular driving behaviors and serve as data sources for establishing pedestrian red-light violation and crossing accident models. Currently, there are the following two main categories of traffic data collection: traditional manual surveys, which can acquire data on vehicle and pedestrian behaviors at intersections via designed questionnaires and data statistics but are time-, resource-, and accuracy-limited, and technologically driven methods, which have gained traction with the advancements in computer and communication technologies, notably, image recognition and processing technologies that automatically recognize video data via computer programs. OpenCV enables the intuitive recognition of road environments and traffic entities, processing data such as traffic flows and entities via visual detection and enhancing traffic management and safety by analyzing and visually presenting the detected data. The OpenCV runtime interface is shown in the following Figure 3.

**Figure 4:**
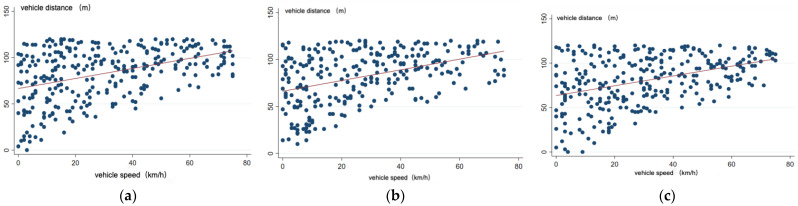
Vehicle speed and distance statistics. (**a**) Statistics of the elderly; (**b**) statistics of middle-aged people; (**c**) statistics of children.

In OpenCV, the following steps are performed for data processing: A video is input for processing, whereby a recorded 24 h video is loaded into YOLO-v5 sequentially and vehicle, pedestrian, and traffic light detection is carried out. The YOLO-v5 network is utilized, and it is trained using the COCO dataset to detect and label targets. For traffic light recognition (i.e., determining the currently illuminated type), a self-designed classification network is employed to detect red, green, and blue in the input traffic light images, and subsequently, target tracking is performed. The target-tracking frame is input, the detected targets are numbered, and then the target speed measurements and distance measurements are carried out. The pixel difference between the tracked targets’ prior and subsequent target frames is selected to calculate the target speed. Finally, data visualization is performed. This process enabled us to recognize and determine intersection vehicle driving behaviors, pedestrian crossing behaviors, and violations. Ultimately, we were able to save the pedestrian crossing features and vehicle driving data results in a CSV table and display the data in the CSV table. A total of 1904 sets of data were collected. To ensure the accuracy of the research results, certain samples were excluded, such as those where age could not be discerned in the video, samples involving patients with disabilities or illnesses, and samples of infants. Consequently, we obtained 1644 valid samples, and the partial numerical statistical results are shown in Table 2 and Figure 4. In the pedestrian age column in Table 2, 0 represents the elderly, 1 represents the middle-aged, and 2 represents children. In the pedestrian crossing choice in Table 2, 0 indicates that traversal was selected, and 1 indicates that traversal was not selected.

## 3. Method

**Figure 5:**
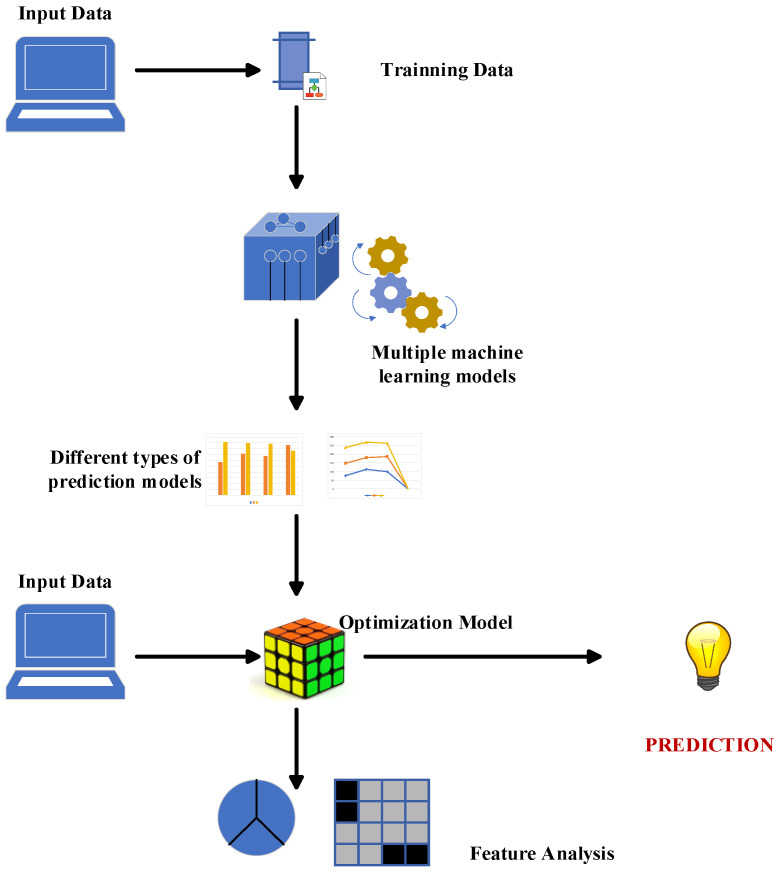
Pedestrian crossing prediction methods and procedures.

This study implemented a machine learning approach to predict pedestrian crossing probability and speed during street crossings, and it proposes a research method that is divided into the following parts: the previous chapter discussed the data collection for the pedestrian crossing features and vehicle driving data results, which led to the acquired dataset. Subsequently, four different machine learning models are established, and each model’s experimental results are displayed herein. The most suitable machine learning model for this research is determined via analysis and comparison. The factors influencing the results are also discussed herein. Figure 5 depicts a schematic diagram of the proposed research method.

### 3.1. Data Preprocessing

After completing the data collection, label encoding was applied to the categorical feature ‘z’ in the obtained dataset, converting it into a numerical representation. The categorical column ‘p’ was transformed into a binary categorical column for the model training. The dataset was then subjected to z-score standardization.

### 3.2. Selection of Machine Learning Models

After appropriate data processing, this study employed four algorithms, namely, decision trees, the Bayesian algorithm, the BP neural network, and a support vector machine, to separately predict crossing probability and crossing speed.

#### 3.2.1. Decision Tree

**Figure 6:**
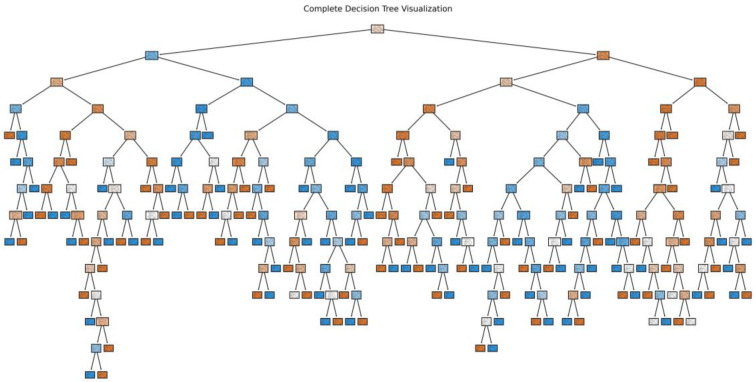
Structure diagram of decision tree.

Decision trees formulate models utilizing tree structures, and they strive to predict numerical outputs via the adoption of straightforward decision rules. A dataset is progressively broken down into smaller subsets while concurrently constructing associated decision trees, as depicted in Figure 6. The end product is a tree featuring decision nodes and leaf nodes, with leaves symbolizing the outcomes, while the decision nodes indicate the points at which the data were partitioned [23]. This model is characterized by its simplicity, ease of interpretation, and ease of implementation.

#### 3.2.2. Bayesian Algorithm

The Bayesian algorithm exhibits a remarkable ability to handle uncertainty and noise, and hence, it is frequently utilized in classification and regression problems, especially in scenarios with limited data. Grounded in Bayesian probability theory, the algorithm is capable of providing explicit estimates of uncertainty. Furthermore, Bayesian models are conveniently able to be updated, and they can progressively assimilate new data to refine the model, proving to be particularly applicable in dynamic environments such as pedestrian street crossings [24,25].

#### 3.2.3. Support Vector Machines

Support vector machines (SVMs) are supervised learning algorithms developed by Vapnik et al. [26] that are founded on statistical learning theory, and they are widely utilized for addressing classification and regression problems. Moreover, they can make accurate predictions even with limited sample data, making them suitable for resolving the issues discussed in this paper. In this type of algorithm, data points are separated with a hyperplane, and kernel functions are utilized to map the data from the input space to a higher-dimensional feature space where it is easier to locate the optimal separating hyperplane. The hyperplane can be represented with the following linear equation:

(1) $$f\left(x\right)=w\cdot x+b$$

where *w* is the weight vector, and *b* is the bias term. The objective of an SVM is to minimize the following objective function to find the maximum margin:

(2) $$\min_{w,b}\frac{1}{2}\|w{\|}^{2}$$

where ||·|| represents the norm, which is subject to the following constraints for each data point *i*:

(3) $$y_{i}\left(w\cdot x_{i}+b\right)\geq 1$$

Herein, *y_i_* represents the class label of the data point *x_i_*. In practical problems, data are often not linearly separable. To handle non-linearly separable data, an SVM employs the so-called ‘kernel trick’, namely, mapping the original data to a higher-dimensional feature space where they are linearly separable via the kernel function *K*(*x*,*x*′). Common kernel functions include the radial basis function (RBF), polynomial kernels, and multilayer perceptron kernels, among others [27]. This study adopted the radial basis function, which is expressed as follows:

(4) $$K\left(x,x^{\prime }\right)=\exp\left(-\gamma \|x-x^{\prime }{\|}^{2}\right)$$

#### 3.2.4. Multi-Layer Perceptron Neural Network

**Figure 7:**
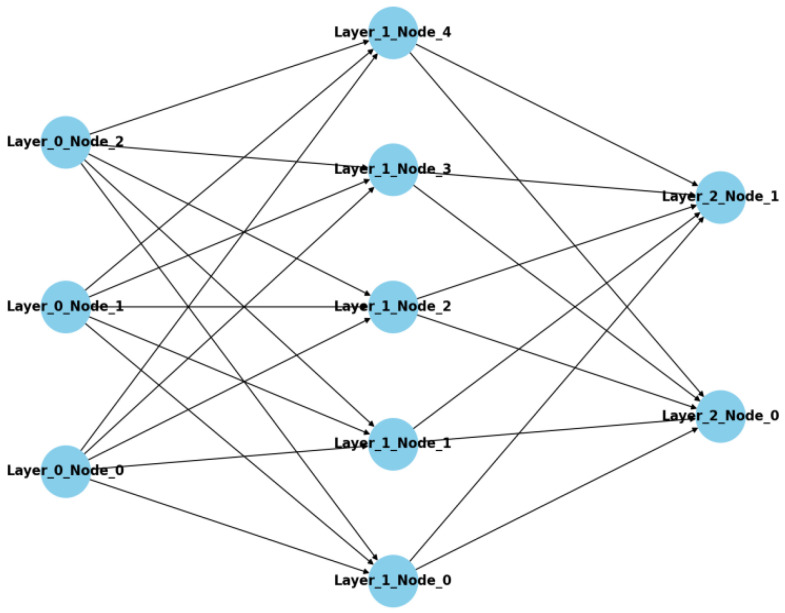
The structure of multi-layer perceptron.

The structural principle of multi-layer perceptrons (MLPs) is illustrated in Figure 7, and they constitute a type of feedforward neural network that encompasses an input layer, hidden layers, and an output layer, with each layer being composed of several neurons that are connected to other layers via weighted connections. An MLP utilizes non-linear activation functions, such as Sigmoid or ReLU, to introduce non-linear properties. Trained via optimization algorithms such as backpropagation and gradient descent, an MLP is capable of learning complex mapping relationships between input data and output labels. Owing to their high flexibility and wide applicability, MLPs are extensively used for classification, regression, and other machine learning tasks.

### 3.3. Parameter Settings

This research employed k-fold cross-validation (k = 5) as the primary hyperparameter-tuning strategy to prevent overfitting. Among various complexity parameters (cp) in the decision tree model, cp = 0.1 outperformed the other values, achieving the highest average performance during the cross-validation process. For the Naïve Bayes algorithm, cross-validation was utilized to select the optimal smoothing parameter α and other parameters. In the support vector machine model (Gaussian kernel), the parameters sigma = 0.01 and C = 0.01 demonstrated superior generalization capabilities during the cross-validation process. In the training of the multi-layer perceptron model, a grid search revealed that a single hidden layer containing 10 neurons exhibited an optimal performance in the model. Sensitivity analysis was also conducted, and although certain parameters (such as the C value in the support vector machine) exhibited relative sensitivities, these sensitivities did not significantly impact the model’s performance within the scope of the current study.

## 4. Results and Discussion

### 4.1. Prediction Results for Crossing Probability

For the prediction of crossing probability, the four aforementioned machine learning models were utilized, with the accuracy, Kappa coefficient, sensitivity, and specificity employed as the evaluation metrics for the models. The results are presented in Table 3.

Based on the results presented, it was observable that the decision tree model exhibited a mediocre predictive performance, with relatively low accuracy and Kappa coefficient. The Naive Bayes model demonstrated moderate performances on both the training and test sets, and despite exhibiting high sensitivity on the training set, it showed lower specificity. The MLP model performed well on both the training and test sets, achieving a higher accuracy and Kappa coefficient. The support vector machine model outperformed the others, offering the highest accuracy and Kappa coefficient, with balanced sensitivity and specificity.

This study also employed techniques such as McNemar’s test for accuracy to statistically test the significance of differences between models. Using a random seed of 42, the results of McNemar’s test for single prediction outcomes among various models are as shown in Table 4.

It can be concluded that there is a significant difference in performance between the MLP and Naïve Bayes models (*p*-value of less than 0.05). This indicates that in this specific scenario, the performances of these two models significantly differ. The *p*-values between some models did not reach the traditional level of significance, but they tended toward significance, for example, “SVM vs. Naive Bayes” and “KNN vs. Naive Bayes”.

To minimize randomness, multiple experiments (20 times) were conducted, each time using a different random seed. The average of the obtained statistical data and *p*-values was then calculated, yielding the following results as shown in Table 5.

It can be inferred that the average *p*-values for most model comparisons are greater than 0.05, indicating no statistically significant difference in performance between the models. However, there is a trend toward significance in the comparisons of “Decision Tree vs. Random Forest”, “SVM vs. Naive Bayes”, “MLP vs. Naive Bayes”, “KNN vs. Naive Bayes”, and “Naive Bayes vs. Random Forest”.

**Figure 8:**
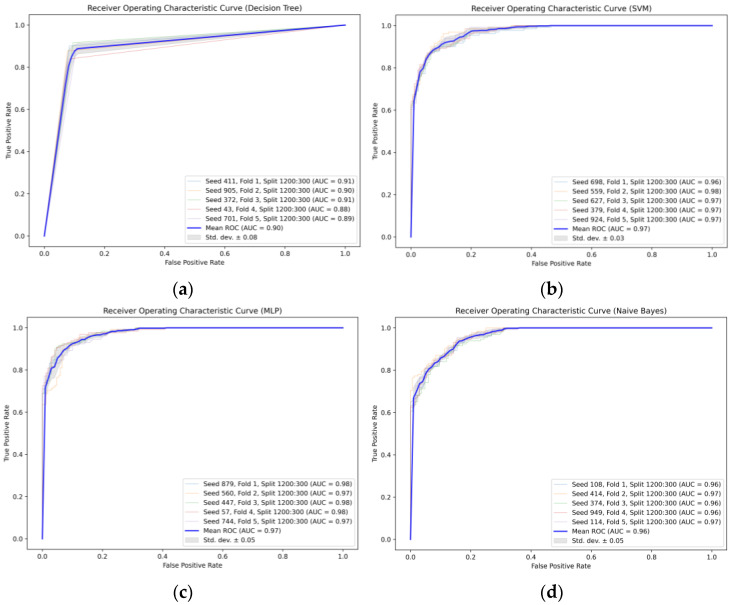
ROC curves for each machine learning model. (**a**) Decision tree; (**b**) SVM; (**c**) MLP; and (**d**) Naïve Bayes.

The ROC curves for each machine learning model are illustrated in Figure 8, where multiple ROC curves, each corresponding to a different cross-validation fold, are plotted in each subplot. The *x*-axis represents the “False Positive Rate (FPR)” while the *y*-axis depicts the “True Positive Rate (TPR)”. The ROC curves were generated by translating the model-predicted probabilities into class labels using varying thresholds. The AUC (area under the curve) is a metric utilized to quantify a model’s performance. Each fold provides an AUC value, which is also displayed on the label of each ROC curve. The average ROC curve, represented in blue, is the mean of the ROC curves across all the folds. The average AUC is the mean value of the AUCs across all the folds. The gray shaded area denotes the standard deviation range of the average ROC curve, providing an indication of the uncertainty in the model’s performance. The red diagonal line illustrates the performance of the random classifier (i.e., a classifier without predictive capabilities), where the true positive rate (TPR) was equal to the false positive rate (FPR). All models used a consistent split ratio (1200:300) to ensure fairness in the comparison of the training and validation set sizes. The variation in the AUC for the same model under different seeds highlights the impacts of the initial conditions and randomness in the model training and validation process.

The Table 6 below shows the dataset split ratios and random seed parameters for each of the four models in different fold tests, along with their corresponding AUC values.

It can be observed that the support vector machine (SVM) and multilayer perceptron (MLP) models consistently exhibit higher AUC values across all folds, indicating superior performance in this specific task. However, considering the other metrics where SVM excels, it is the most suitable model when evaluated comprehensively.

### 4.2. Prediction Results for Crossing Speed

For the prediction of crossing speed, the aforementioned four machine learning models were employed, and we utilized MSE, RMSE, R^2^, and MAD as the evaluation metrics. The performance metrics for each fold are presented in Table 7.

The average performance metrics of the four machine learning models are shown in Table 8.

Based on the aforementioned results, the following conclusions can be drawn: The decision tree model exhibited an average performance on both the training and testing sets, characterized by a higher R^2^, but also a higher MSE, RMSE, and MAD. The naive Bayes model demonstrated the poorest performance on both sets, characterized by the highest MSE, the highest RMSE, the lowest R^2^, and a higher MAD. The MLP neural network model also showed general performance on both sets, associated with its higher MSE, higher RMSE, lower R^2^, and higher MAD. The support vector machine model performed the best, boasting the lowest mean squared error (MSE) and root mean squared error (RMSE), as well as the highest coefficient of determination (R^2^) and a lower mean absolute deviation (MAD).

Overall, the support vector machine model showcased superior performance on this dataset, having the smallest mean squared error and root mean squared error, and it was able to fit the data well, with a relatively high coefficient of determination (R^2^). It can be concluded that the support vector machine algorithm emerges as the most apt model for predicting the probability and speed of pedestrian crossings.

### 4.3. Feature Analysis

#### 4.3.1. Analysis of Pedestrian Crossing Probability

To investigate the impacts of various features on prediction outcomes, a feature importance ranking analysis was conducted using permutation importance. Permutation importance is a method to assess the impacts of features on the performance of prediction models, primarily based on the idea of shuffling the values of each feature and observing the effect of this disruption on the model performance. Unlike other feature importance methods, Permutation importance is model-agnostic, meaning it can be used with any model. Initially, the model is trained using the original dataset, and its performance is evaluated. Then, for each feature, the values in that feature column are randomly shuffled, keeping other features unchanged, and the performance is reassessed to record the change in the model performance. The permutation importance of a feature is typically defined as the difference between the original performance and the performance after shuffling that feature. If shuffling a particular feature significantly decreases model performance, this implies that the feature is very important for model predictions. To minimize the impact of randomness, this process is repeated multiple times, and the average change in performance is taken as the permutation importance of that feature. Upon analyzing the permutation importance of each feature in the best-performing SVM model, the results are as follows: the importance of feature x is 0.299, y is 0.151, and z is 0.002. Hence, it can be concluded that x has the most significant impact on the model, followed by y, while the impact of feature z is relatively minor.

**Figure 9:**
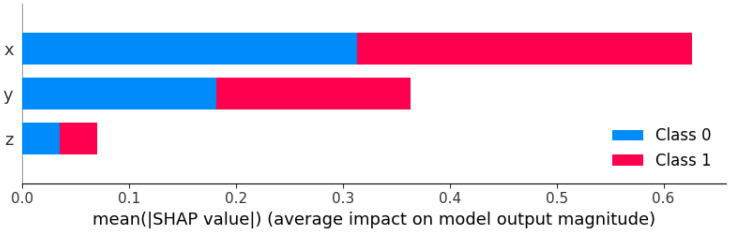
SHAP analysis conducted on the crossing probability prediction model based on the SVM.

After evaluating the feature importance for the entire model, we conducted a Shapley additive explanations (SHAP) analysis on the SVM-based cross-probability prediction model for a deeper analysis of individual predictions, as shown in Figure 9.

From the graphical analysis, it is evident that the ‘x’ feature exerted the most substantial impact on the predictive outcomes, where an increase in its value inclined the model toward predicting p. The influence of the ‘y’ feature was secondary, with its value increase also biasing the model toward a p prediction. The ‘z’ feature exhibited a relatively minimal impact on the classification results. Consequently, the vehicle speed surface was the paramount factor affecting a pedestrian’s decision to cross, followed by the vehicle distance. Although the age factor held a relatively modest influence, it still imparted a discernible impact on the predictive values.

**Figure 10:**
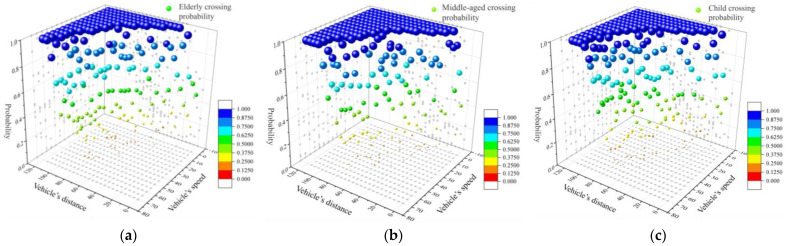
Probability model of pedestrians’ crossing behaviors. (**a**) Crossing probability model for the elderly; (**b**) crossing probability model for middle−aged adult pedestrians; (**c**) crossing probability model for children.

To explore the impact patterns of various factors across different age groups on the predictive outcomes, this study separately visualized the output results of the SVM-based crossing probability prediction model for different age segments. Figure 10 illustrates a three-dimensional scatter plot established based on vehicle speed, vehicle distance, and pedestrian crossing probability. The points in the graphs represent the probabilities of pedestrians opting to jaywalk with different vehicle distances and speeds. Distances are categorized into 24 groups, ranging from 0–5 m to 115–120 m, while speeds are divided into 15 groups, from 0–5 km/h to 70–75 km/h. Each increment in distance or speed represents an increase to the next interval in its respective range. The graph reveals that the probability of a pedestrian choosing to cross increased with the enlargement of the nearest vehicle’s distance, and it decreased with an escalation in the nearest vehicle’s speed.

The crossing probability model for the elderly is illustrated in Figure 8a. As the vehicle speed increased, the likelihood of an elderly pedestrian choosing to cross decreased, and conversely, as the vehicle distance increased, an elderly pedestrian’s crossing probability increased. When the nearest vehicle on the roadway was more than 95 m away, the elderly were nearly 100% likely to choose to cross, regardless of the vehicle’s speed. The mapping distribution of the elderly pedestrians’ crossing probabilities with respect to vehicle distance leaned left compared with those of middle-aged adult and child pedestrians, indicating that the overall vehicle distance was greater when an elderly pedestrian chose to cross. The crossing probability model for middle-aged adult pedestrians is depicted in Figure 8b. With an acceleration in vehicle speed, the probability of a middle-aged individual jaywalking diminished, while with an extension in vehicle distance, their jaywalking probability was augmented. When the distance to the nearest vehicle on the lane exceeded 75 m, the likelihood of a middle-aged individual choosing to cross was approximately 100%, regardless of the vehicle’s speed. The distribution of the middle-aged adult pedestrians’ crossing probability model, in relation to vehicle distance, was generally right-aligned compared with those of elderly and child pedestrians, implying that middle-aged adults were willing to cross at larger vehicle distances. Adults, having a heightened perception of vehicle speed and distance, along with abundant street-crossing experience, tended to take more risks when crossing. The children’s crossing probability model, as shown in Figure 8c, demonstrated a decrease in the likelihood of running a red light with an increase in vehicle speed, whereas an augmentation in the crossing probability occurred as vehicle distances grew. Once the vehicle in the closest lane was more than 80 m away, regardless of its speed, a child was nearly 100% likely to opt to cross. The mapping distribution of the crossing probability model for children concerning vehicle distance presented considerable variability, indicating a substantial discreteness in crossing probability. Children, with their relatively limited cognition of vehicle speed and distance and their lack of street-crossing experience, exhibited a more scattered mapping for speed and distance.

#### 4.3.2. Analysis of Pedestrian Crossing Speed

To iIn the regression analysis, the SVR model exhibited the most favorable performance. Feature importance was ranked within this model, revealing that the ‘x’ feature holds the highest importance with a score of 1.03, followed by ‘y’ with a score of 0.81, while the importance of ‘z’ is comparatively lower at 0.16.

**Figure 11:**
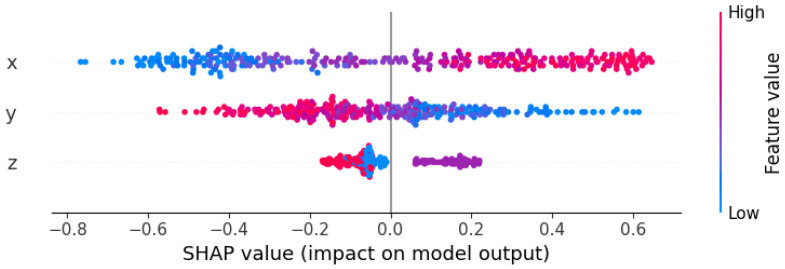
SHAP analysis based on the support vector regression (SVR) model.

After evaluating the feature importance for the entire model, we conducted a Shapley additive explanations (SHAP) analysis on the SVM-based cross-probability prediction model for a deeper analysis of individual predictions, as shown in Figure 11.

In the SHAP plot, red indicates the higher feature values, while blue denotes the lower ones. The diagram reveals that the x feature was the most crucial, y was the second most vital, and z had a comparatively minor impact. For the x feature, higher values (shown in red) were associated with larger positive SHAP values, while lower values (shown in blue) correlated with more significant negative SHAP values, indicating a positive correlation between the x values and the predictions. Higher values for y (shown in red) correlated with negative SHAP values, signifying that the y values were inversely related to the predictions. The SHAP values for the z feature were relatively evenly distributed, with many colors mixed together (i.e., a blend of red and blue points), which could imply that the relationship between this feature and the prediction outcome was non-linear or more complex.

**Figure 12:**
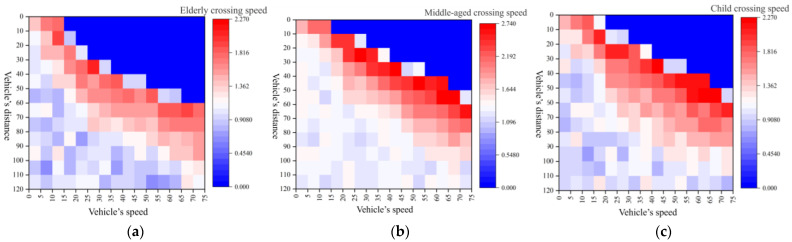
Crossing speed model of the pedestrians. (**a**) Crossing speeds of elderly individuals; (**b**) crossing speeds of middle-aged individuals; (**c**) crossing speeds of children.

Upon analyzing the influence of each feature on the predictive model, this study further visualized the output results of the crossing speed model for the different age groups, as depicted in Figure 12. Here, the deeper red areas represent faster pedestrian crossing speeds while the deeper blue areas indicate slower speeds.

Figure 10a illustrates the crossing speeds of elderly individuals. The points (15, 0), (75, 60), and (75, 0) generated a dark blue, triangular-like region for the elderly, wherein the probability of crossing within this interval was zero, and thus the speed was also zero. The red area denotes scenarios where the elderly had limited time to cross, necessitating a swifter crossing speed. The white area represents scenarios where pedestrians had ample time to cross, and thus, they opted to traverse at normal speeds. Due to the physical constraints that align with age, the crossing speeds of the elderly were slower than those of younger adults. Figure 10b presents the speed distributions of the young and middle-aged individuals choosing to cross at different vehicle speeds and distances. The young and middle-aged individuals formed a dark blue, triangular-like region at points (15, 0), (75, 50), and (75, 0). Due to excessively high vehicle speeds or minimal vehicle distances, the probability of crossing within these intervals was zero for young and middle-aged individuals. With a more pronounced inclination toward risk taking, the overall deep red area was more prevalent, signifying a general preference amongst young and middle-aged individuals to engage in riskier, faster crossings. Figure 10c depicts the crossing speed distributions for children based on varying vehicle speeds and distances. At points (15, 0), (75, 50), and (75, 0), the children formed a dark blue, triangular-like area, wherein the crossing probability was zero due to either overly high vehicle speeds or insufficient vehicle distances. Previous analyses have illuminated that children due to inaccurate judgments about vehicle speeds and distances and limited crossing experience, exhibit dispersed crossing probabilities. Coupled with the widespread red area in the current speed distribution depiction, it was evident that even when the vehicle distance and speed would permit crossing at regular speeds, children were predisposed to choose faster crossing speeds during traversal.

### 4.4. Application Method

Simulating realistic pedestrian crossing decisions enhances the pedestrian module’s application in traffic simulation software. PTV−VISSIM2020, a leading microsimulation software, facilitates the construction of complex traffic environments. In Vissim, pedestrian simulation is crucial for assessing the pedestrian flow capacity and infrastructure service levels, yet it overlooks pedestrian safety evaluation. The pedestrian module in Vissim, operating in a social force model, is ‘repulsive’ to the vehicle module and assumes strict adherence to traffic signals by both pedestrians and vehicles, limiting the evaluation of safety in current traffic environments and the ‘realistic’ simulation of traffic entities. Future developments in the Vissim platform could involve modifying the pedestrian module for a more authentic simulation of pedestrian crossing behaviors, considering varied pedestrian and vehicle characteristics.

## 5. Conclusions and Future Prospects

This study, conducted using real-world data collected from four signal-controlled intersections in Dalian, China, uniquely applied a variety of machine learning methods to evaluate, analyze, and predict jaywalking behaviors at pedestrian crosswalks. The methods included Bayesian models, decision trees (DTs), support vector machines (SVMs), and multilayer perceptrons (MLPs). Precise pedestrian crosswalk and vehicle motion data were extracted using OpenCV technology, providing high-quality inputs for the machine learning models. This study highlighted the compensatory nature of the SVM model in predicting crossing probabilities, exhibiting outstanding performance in metrics such as accuracy, Kappa coefficient, sensitivity, and specificity. Additionally, techniques like McNemar’s test for accuracy were employed to statistically test the significance of differences between models. Feature importance ranking, SHAP analysis, and feature quantity visualization were used to analyze the SVM model from different aspects. This work provides significant technical support for pedestrian safety in the intelligent vehicle domain, especially in terms of predicting pedestrian behavior and devising accident prevention measures.

However, the data in this study are from major cities in China, and the results may not fully apply to traffic environments under different national regulations or settings. Moreover, the study only addressed conflicts between motor vehicles and pedestrians in predicting pedestrian crossing behavior and did not investigate the specific impacts of non-motor vehicles and public buses on such behaviors.

Future work could expand the scope of the data collection to include various types of vehicles (like buses, bicycles, trucks, etc.) and more diverse urban environments. A deeper investigation into other factors influencing pedestrian crosswalk behavior, integrating these as machine learning features, could further enhance model accuracy and practicality. Our plans are to apply these findings to existing traffic simulation systems, such as VISSIM, SUMO, and the Transportation Modeling Platform, for a more realistic simulation of pedestrian behaviors, thereby enhancing their roles in intelligent transportation systems. Additionally, the development of a machine learning model capable of real-time prediction of pedestrian jaywalking behavior, to be implemented in intelligent vehicles, is also underway.

## Figures and Tables

**Table 1:** Statistics.

Type	Date	Location	Collection Time	Duration
8-lane roadway	14 November 2022	Zhangqian Road–Hongjin Road	6:45–9:45	180 min
			12:00–15:00	180 min
	15 November 2022	Huadong Road–Qianshan Road	7:00–10:00	180 min
			12:00–15:00	150 min
6-lane roadway	15 November 2022	Shandong Road–Songjiang Road	6:45–9:45	180 min
			11:30–2:00	150 min
	14 November 2022	Hongyun Road–Zhelin Street	7:00–10:00	180 min
			16:00–19:00	180 min

**Table 2:** Statistics on vehicle speed (x), vehicle distance (y), pedestrian age (z), pedestrian crossing choice (p), and crossing speed (v).

x (km/h)	y (m)	z	p	v (m/s)
13	37	1	1	0
82	58	2	1	0
49	29	2	1	0
5	23	0	1	0
86	48	1	1	0
115	15	1	0	1.26
109	26	2	0	1.18
…				

**Table 3:** Prediction results for the machine learning models.

Technique	Accuracy (%)	Kappa Coefficient	Sensitivity (%)	Specificity (%)
Decision tree	92	0.637	81.7	82.4
Bayesian	94.3	0.788	96.4	83.2
MLP	92	0.717	98.4	77.0
SVR	92	0.813	92.4	88.8

**Table 4:** Model comparison in single experiment.

Model Comparison	Statistical Data	*p*-Value	Significance Analysis
Decision tree vs. SVM	0.696	0.404	No significant difference
Decision tree vs. MLP	1.44	0.230	No significant difference
Decision tree vs. Naïve Bayes	0.121	0.728	No significant difference
SVM vs. MLP	0.167	0.683	No significant difference
SVM vs. Naïve Bayes	2.722	0.099	There is no significant difference, but it tends to be significant, which is worth further exploration
MLP vs. Naïve Bayes	4.5	0.034	There is a significant difference, indicating that the performance of the two models is significantly different

**Table 5:** Model comparison across multiple experiments.

Model Comparison	Average Statistical Data	Average *p*-Value	Significance Analysis
Decision tree vs. SVM	2.694	0.331	No significant difference
Decision tree vs. MLP	2.177	0.318	No significant difference
Decision tree vs. Naive Bayes	0.816	0.537	No significant difference
SVM vs. MLP	1.064	0.502	No significant difference
SVM vs. Naive Bayes	4.820	0.126	There is no significant difference, but it tends to be significant, which may indicate that there are performance differences between models
MLP vs. Naive Bayes	3.009	0.284	There is no significant difference, but it tends to be significant and may need more data verification

**Table 6:** AUC values of each model.

Model	Fold	Seed	Split Ratio	AUC
Decision tree	1	596	1200:300	0.881764
	2	977	1200:300	0.882486
	3	565	1200:300	0.880411
	4	90	1200:300	0.873843
	5	513	1200:300	0.865351
SVM	1	51	1200:300	0.975063
	2	379	1200:300	0.980226
	3	924	1200:300	0.976258
	4	558	1200:300	0.969963
	5	397	1200:300	0.973170
MLP	1	206	1200:300	0.973259
	2	424	1200:300	0.968795
	3	261	1200:300	0.980294
	4	77	1200:300	0.968291
	5	879	1200:300	0.971182
Naive Bayes	1	545	1200:300	0.958243
	2	541	1200:300	0.971884
	3	788	1200:300	0.973530
	4	995	1200:300	0.962103
	5	631	1200:300	0.977370

**Table 7:** The performance metrics for each fold.

Model	Fold	Random Seed	Train Ratio	Test Ratio	MSE	RMSE	MAD	R^2^
Decision tree	1	6132	0.8	0.2	0.225215	0.474568	0.430690	0.478519
	2	7321	0.8	0.2	0.199337	0.446472	0.410487	0.510846
	3	5204	0.8	0.2	0.190829	0.436840	0.397907	0.531904
	4	43	0.8	0.2	0.209782	0.458020	0.411726	0.512356
	5	1975	0.8	0.2	0.176267	0.419842	0.392056	0.577930
Bayesian Ridge	1	114	0.8	0.2	0.212515	0.460993	0.377595	0.462405
	2	3610	0.8	0.2	0.246985	0.496975	0.397476	0.410508
	3	7240	0.8	0.2	0.260120	0.510020	0.413945	0.388587
	4	5365	0.8	0.2	0.255769	0.505736	0.399616	0.393398
	5	2144	0.8	0.2	0.280739	0.529848	0.426510	0.348115
SVR	1	7544	0.8	0.2	0.210965	0.457642	0.423549	0.516097
	2	9700	0.8	0.2	0.202718	0.450198	0.439083	0.529888
	3	4056	0.8	0.2	0.124318	0.473622	0.375354	0.639779
	4	6671	0.8	0.2	0.200251	0.447952	0.374838	0.496490
	5	7528	0.8	0.2	0.228423	0.373082	0.401708	0.483284
MLP	1	4934	0.8	0.2	0.195281	0.441907	0.395040	0.547751
	2	3821	0.8	0.2	0.179646	0.423847	0.389555	0.555520
	3	90	0.8	0.2	0.207948	0.456013	0.403807	0.509789
	4	9156	0.8	0.2	0.189576	0.435404	0.390474	0.519733
	5	9706	0.8	0.2	0.245861	0.495844	0.419475	0.437373

**Table 8:** The average performance of each model.

Model	MSE	RMSE	MAD	R^2^
Bayesian Ridge	0.251226	0.500715	0.403028	0.400602
Decision tree	0.200286	0.447148	0.408573	0.522311
MLP	0.203663	0.450603	0.397670	0.514033
SVM	0.193335	0.440499	0.402906	0.533108

## Data Availability

Data are contained within the article.
